# Sex-related differences in platelet aggregation

**DOI:** 10.1093/ehjcvp/pvaf052

**Published:** 2025-07-17

**Authors:** Marco Cattaneo

**Affiliations:** Dipartimento di Scienze della Salute, Università degli Studi di Milano, 20142 Milano, Italy; Fondazione Arianna Anticoagulazione, Via Paolo Fabbri 1/3, 40138 Bologna, Italy

**Keywords:** Sex, Platelet aggregation, Aspirin, Clopidogrel, Haematocrit, Ionized calcium, Laboratory artefacts

The recently published study by Galli *et al*.^1^ shows that, when platelet aggregation is measured by light-transmission aggregometry (LTA) in platelet-rich plasma (PRP) anticoagulated with citrate, the extent of platelet aggregation induced by ADP is higher in women than men.^1^ The authors should be commended for their study, which is among the largest ones published on this topic, as it included 1483 subjects not on antiplatelet therapy (428 without cardiovascular risk factors), studied in a single specialized academic centre. Very similar results were described by previous reports published in the last 50 years, including the two large epidemiological studies Northwich Park Heart Study^2^ and Framingham Study.^3^ In 1980, Kelton *et al.*^4^ demonstrated that the higher *in vitro* aggregation response of women’s platelets is actually caused by an artefact: When blood is collected in the standard amount of citrate (9:1, vol:vol, blood:anticoagulant), the lower haematocrit levels of women compared with men increases the volume distribution of citrate, resulting in higher plasma Ca^2+^ concentrations, which support increased platelet aggregation *in vitro*.^4^ Indeed, when blood samples were collected in citrate volumes that had been adjusted for the haematocrit according to a mathematically-derived standard curve, differences between women and men disappeared.^4^ These results were replicated in subsequent studies comparing standard vs. haematocrit-adjusted citrate concentrations^5^ and are compatible with the observation that platelet ‘hyper-aggregability’ in women was observed in populations in which women had lower haematocrit levels than men,^2,3,6,7^ but not in men and women with similar haematocrit.^6^

In our experience, the extent of platelet aggregation measured by LTA is higher in women than men when studied in citrate-anticoagulated PRP, but similar in women and men when tested in hirudin-anticoagulated PRP, where the concentration of Ca^2+^ is not decreased by the anticoagulant. Hirudin is the anticoagulant of choice for measuring platelet aggregation in whole blood samples by the impedance aggregometry-based Multiplate™. However, platelet aggregation was still higher in women’s hirudin-anticoagulated PRP when tested by Multiplate™, but the difference with men’s PRP in this case was due to women’s higher platelet count,^8^ which artifactually affects the results of platelet aggregation measured by Multiplate™.^9^ Therefore, there is strong experimental evidence that enhanced *in vitro* platelet aggregability in women is consequent to artefacts, rather than reflecting a true, biologically relevant phenomenon. A study that confirmed the ‘hyper-aggregability’ of women’s citrate-PRP concluded that men’s blood actually shows enhanced platelet-dependent primary haemostatic activity when tested by different platelet function tests, such as the bleeding time and platelet interaction with arterial subendothelium.^7^

Galli *et al*.^1^ also addressed the important point of sex differences in the results of platelet aggregation monitoring in 4204 patients on antiplatelet therapy with aspirin, clopidogrel, or dual antiplatelet therapy with aspirin plus clopidogrel. Monitoring the effects of treatments has historically been an important strategy in medicine, aiming at increasing drug efficacy and safety by tailoring the intensity and/or type of therapy for poor-responders or hyper-responders. This strategy has been applied to any therapeutic intervention that modifies measurable parameters, such as drugs decreasing blood pressure, drugs decreasing blood concentrations of glucose or cholesterol, drugs inhibiting blood coagulation (vitamin K antagonists, unfractionated heparin), and many others. Attempts to monitor antiplatelet therapy have been made only at the beginning of the 3rd millennium, based on the demonstration of high inter-individual variability of response, mainly to clopidogrel. Randomized clinical trials performed in Western countries failed to show any clinical benefit of monitoring antiplatelet therapy with platelet function tests.^10^ This failure is due to instability of the platelet reactivity phenotype, lack of standardisation and, perhaps more importantly, imprecision and inaccuracy of the platelet aggregation tests, which are affected by potential artefacts.^10^ Not surprisingly, in the study by Galli *et al*.^1^ the extent of platelet aggregation during antiplatelet therapy was higher in women than men, because the artefact caused by citrate affects the results independently of whether or not the aggregability of platelets has been inhibited by drugs. As a consequence, women were perhaps inaccurately considered less responsive to antiplatelet agents than men in the study by Galli *et al*.^1^

Based on these considerations, I think that it would be interesting to know whether the observed sex difference in the study by Galli *et al*.^1^ persisted after restriction of their analysis to women and men well matched for haematocrit levels.

## Data Availability

No data were generated or analysed for this article.
